# Clinical outcome of ampicillin or ampicillin/sulbactam versus glycopeptides in ampicillin-susceptible *Enterococcus faecalis/faecium* bacteremia: a 10-year retrospective cohort study

**DOI:** 10.1186/s12879-024-09824-w

**Published:** 2024-09-02

**Authors:** Yeol Jung Seong, Je Eun Song, Eugene Lee, Eun Jin Kim, Jung Yeon Heo, Young Hwa Choi, Yong Chan Kim

**Affiliations:** 1 Department of Internal Medicine, Busan Medical Center, Busan, Korea; 2 Department of Internal Medicine, Inje University Ilsan Paik Hospital, Goyang, Korea; 3 Department of Biology, Duke University, Durham, NC USA; 4 Department of Infectious Diseases, Ajou University School of Medicine, 164 World cup-ro, Yeongtong- gu, Suwon, 16499 Republic of Korea; 5 Division of Infectious Diseases, Department of Internal Medicine, Yongin Severance Hospital, Yonsei University College of Medicine, 363 Dongbaekjukjeon-daero, Giheung-gu, Yongin-si, Gyeonggi-do 16995 Republic of Korea

**Keywords:** Bacteremia, *Enterococcus faecalis*, *Enterococcus faecium*, Anti-bacterial agents, Mortality

## Abstract

**Background:**

Glycopeptides for ampicillin-susceptible *Enterococcus faecalis/faecium* bacteremia are readily prescribed depending on the severity of the condition. However, there is limited data on the outcomes of glycopeptide use compared to ampicillin-containing regimens for ampicillin-susceptible *E. faecalis/faecium* bacteremia. From an antibiotic stewardship perspective, it is important to determine whether the use of glycopeptides is associated with improved clinical outcomes in patients with ampicillin-susceptible *E. faecalis/faecium* bacteremia.

**Methods:**

This retrospective cohort study was conducted at a university-affiliated hospital between January 2010 and September 2019. We collected data from patients with positive blood cultures for *Enterococcus* species isolates. The clinical data of patients who received ampicillin-containing regimens or glycopeptides as definitive therapy for ampicillin-susceptible *E. faecalis/faecium* bacteremia were reviewed. Multivariate logistic regression analysis was performed to identify risk factors for 28-day mortality.

**Results:**

Ampicillin-susceptible *E. faecalis/faecium* accounted for 41.2% (557/1,353) of enterococcal bacteremia cases during the study period. A total of 127 patients who received ampicillin-containing regimens (*N* = 56) or glycopeptides (*N* = 71) as definitive therapy were included in the analysis. The 28-day mortality rate was higher in patients treated with glycopeptides (19.7%) than in those treated with ampicillin-containing regimens (3.6%) (*p* = 0.006). However, in the multivariate model, antibiotic choice was not an independent predictor of 28-day mortality (adjusted OR, 3.7; 95% CI, 0.6–23.6).

**Conclusions:**

Glycopeptide use was not associated with improved mortality in patients with ampicillin-susceptible *E. faecalis/faecium* bacteremia. This study provides insights to reduce the inappropriate use of glycopeptides in ampicillin-susceptible *E. faecalis/faecium* bacteremia treatment and promote antimicrobial stewardship.

## Introduction

*Enterococcus* species can cause a variety of nosocomial infections, including urinary tract infections, intra-abdominal infections, infective endocarditis, surgical wound infections, bacteremia, and neonatal infections [1–3]. Enterococci are the third most common etiological bacteria isolated from patients with nosocomial bacteremia [4]. Physicians prioritize the management of enterococcal bacteremia due to its high mortality rate, particularly in critically ill patients in intensive care units (ICU) [5].

Currently, there are 17 known *Enterococcus* species, but only a few causes clinical infections in humans. The most common and important species within the genus *Enterococcus* are *E. faecalis* and *E. faecium*. Historically, most enterococcal infections have been caused by *E. faecalis*, but since the early 1990s, there has been a steady increase in *E. faecium* infections [1]. Currently, these two strains have similar incidence rates, but *E. faecium* is remarkably more resistant to vancomycin and ampicillin. Consequently, clinicians may find it difficult to determine whether to use glycopeptide- or ampicillin-containing regimens for the treatment of enterococcal bacteremia before the susceptibility results are known. In addition, in some cases, glycopeptides are retained as definite therapy even after the identification of ampicillin-susceptible isolates in susceptibility tests [6], especially when treating critically ill patients.

Only a few studies have compared the clinical outcomes of ampicillin-containing antibiotics and glycopeptides in the treatment of ampicillin-susceptible enterococcal bacteremia [7–9]. An in vitro study on *Enterococcus* strains demonstrated that beta-lactam antibiotics such as ampicillin exhibit greater activity and lower minimum inhibitory concentrations (MIC) than vancomycin [10]. In another study conducted in Australia in 2014, glycopeptide use was associated with increased mortality in patients with *E. faecalis* bacteremia [9]. Enhancing antimicrobial stewardship requires further investigation into the need for glycopeptides for the treatment of ampicillin-susceptible *E. faecalis/faecium* bacteremia.

Therefore, in alignment with antimicrobial stewardship principles, in this study, we investigated the effects of glycopeptide- and ampicillin-containing antibiotic therapy in ampicillin-susceptible *E. faecalis/faecium* bacteremia on clinical outcomes.

## Methods

### Study design and population

This retrospective study was conducted in a 1,108-bed university-affiliated hospital between January 2010 and September 2019. We accessed the data for research purpose in March 2022. All patients from whom clinical isolates of *E. faecalis* and *E. faecium* were obtained from blood cultures were included in this study. Blood cultures were processed using an automated system. The BACTEC FX system (BD Diagnostic Systems, USA) and BacT/ALERT 3D system (bioMérieux, Durham, NC, USA) were used for microbial detection. Antimicrobial susceptibility testing was performed using the MIC agar dilution method, as described in the Clinical and Laboratory Standards Institute (CLSI) M100 guidelines.

The following cases were excluded from the study: (1) age < 18 years, (2) expiration within 7 days, (3) polymicrobial bacteremia, (4) use of definite antibiotics for less than 3 days, (5) use of empiric antibiotics for > 5 days, and (6) longer duration of empiric antibiotic use than definite antibiotic use. Patients were divided into the ampicillin-containing group and the glycopeptide group based on the definite antibiotics used after susceptibility results were known. The ampicillin-containing group was treated with ampicillin or ampicillin/sulbactam as definitive antibiotics. The glycopeptide group received vancomycin or teicoplanin as definitive therapy.

### Data collection and definition

Data on demographic characteristics, underlying diseases, immunocompromised status, primary site of infection, site of acquisition, duration of antibiotic use, and outcomes were collected from electronic medical records. In addition, the Pitt Bacteremia Score, which ranges from 0 to 14, was used to assess the severity of illness caused by bacteremia [11–13].

*E. faecalis/faecium* bacteremia was defined as the presence of *E. faecalis or E. faecium* in one or more blood cultures. Recurrence of bacteremia was defined as the re-detection of *E. faecalis/faecium* from a blood culture test within 30 days of the identification of negative blood culture results. Persistent bacteremia was defined as the isolation of *E. faecalis/faecium* from blood cultures 7 days after effective antibiotic therapy. Hospital-acquired infection was defined as bacteremia that occurs ≥ 48 h after admission, or bacteremia in which the patient had been hospitalized at any time within the previous month. Empiric and definite antibiotics were defined as antibiotics used before and after the determination of susceptibility profiles, respectively.

The primary endpoint of the current study was 28-day all-cause mortality. Secondary endpoints included 30-day recurrence of bacteremia, persistent bacteremia, adverse drug events, duration of hospitalization after bacteremia, and ICU admission after bacteremia.

### Statistical analysis

All statistical analyses were performed using SPSS version 28 for Windows (IBM Corp., Armonk, NY, USA). Continuous variables were compared using two-sample t-tests or Wilcoxon rank-sum tests. Categorical variables were evaluated using Chi-square tests or Fisher’s exact tests. Significant variables (*P*-values of < 0.05) were further subjected to multivariable logistic regression analysis for their association with 28-day mortality.

## Results

### Epidemiology of *E. faecalis/faecium* bacteremia

**Fig. 1:**
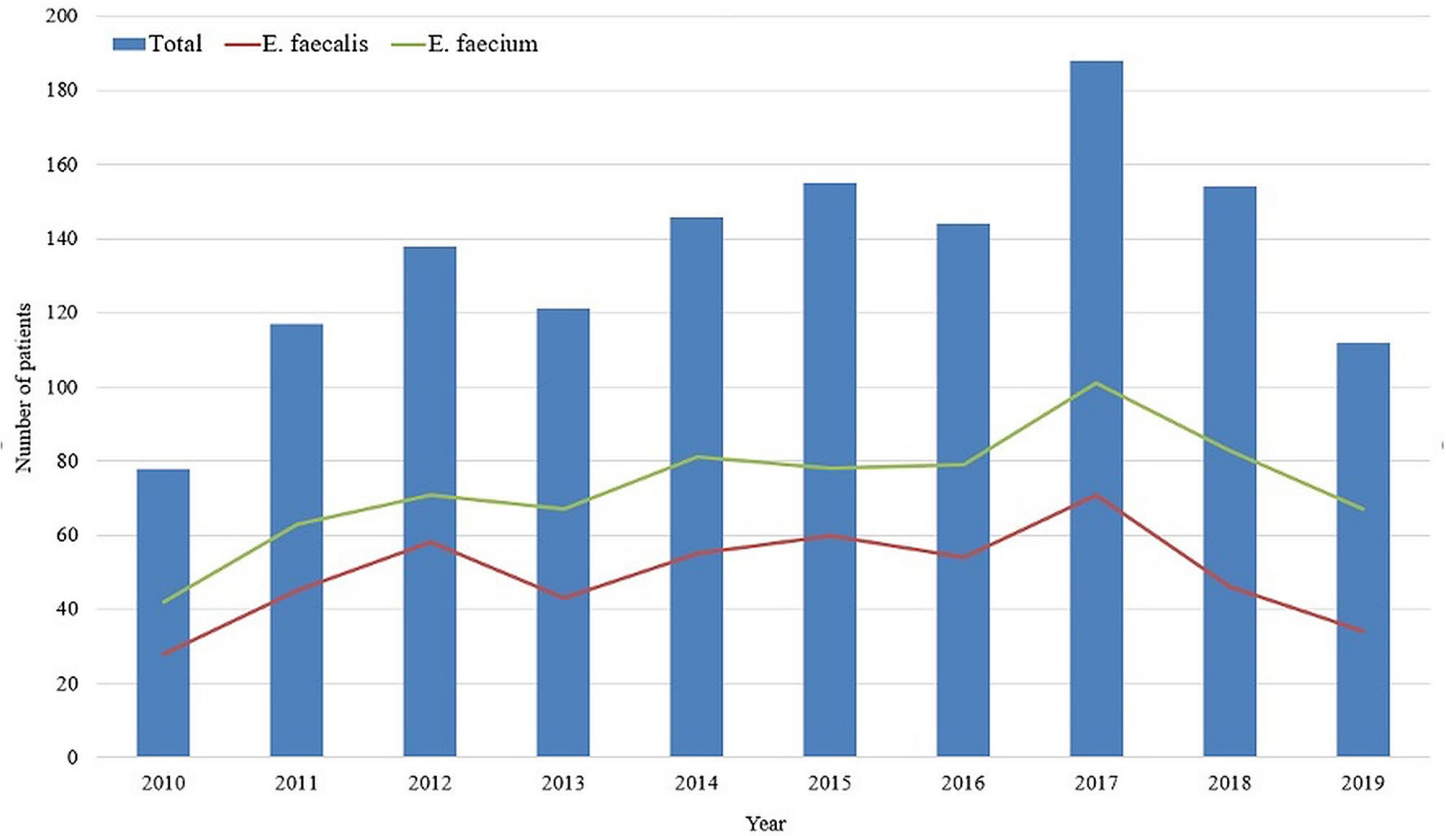
Incidence of Enterococcal bacteremia from 2010 to 2019

Over the 10-year study period, 1,353 patients with enterococcal bacteremia were identified; the annual incidence of enterococcal bacteremia infection is presented in Fig. 1. Of all *Enterococcal* species detected, 1211 (89.6%) were *E. faecalis/faecium*. *E. faecalis* was detected in 494 cases and *E. faecium* in 732 cases, while 15 cases exhibited *E. faecalis* and *E. faecium* co-infection. Most *E. faecalis* isolated from bacteremia were ampicillin-susceptible strains (97.4%, 481/494), whereas only 12.7% (93/732) of *E. faecium* bacteremia were ampicillin-susceptible strains. Vancomycin-resistant enterococci (VRE) accounted for 20.3% (246/1211) of *E. faecalis/faecium* bacteremia cases.

### Comparison of ampicillin-containing and glycopeptide treatment groups

**Fig. 2:**
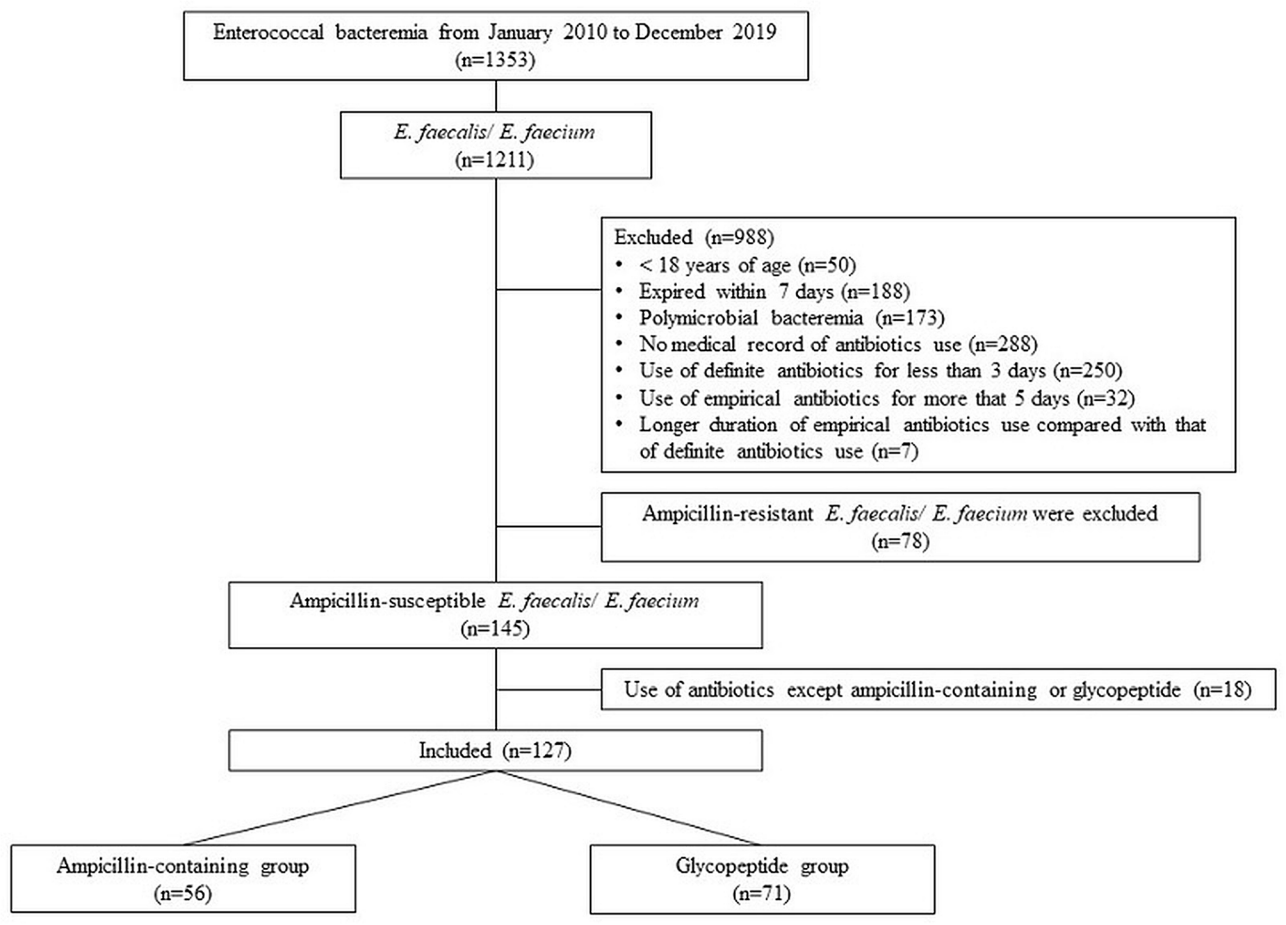
Flow chart of the patient enrollment

Of the 1211 patients with *E. faecalis* and *E. faecium* bacteremia, 127 met the inclusion criteria (Fig. 2). Ampicillin-containing antibiotics and glycopeptides were the definitive antibiotics for 56 (44.1%) and 71 (55.9%) patients, respectively. The patient demographics are shown in Table 1. The median age of the patients was 64.3 ± 14.62 years, and 36.2% of the patients were female. There were no significant differences in underlying medical conditions between the two groups, except for a higher prevalence of chronic kidney disease in the ampicillin-containing group. There was a higher proportion of immunosuppressed patients in the glycopeptide group than ampicillin or ampicillin/sulbactam group, particularly those undergoing chemotherapy, though this was not significantly different (21.1% vs. 8.9%, *p* = 0.061). The Pitt Bacteremia Score and rate of hospital-acquired infection were higher in the glycopeptide group, and the *P*-value for each independent variable was < 0.001. The urinary tract was the most common site of infection, and urinary tract infections accounted for 25.2% (32/127) of all cases. Within the treatment groups, catheter-related infection was the leading cause of bacteremia in the glycopeptide group, accounting for 26.8% (19/71) of cases, whereas urinary tract infection was the leading cause of bacteremia in the ampicillin-containing group, accounting for 37.5% (21/56) of cases. The duration of antibiotic use was 2.71 ± 1.209 days for empiric antibiotics and 12.02 ± 10.308 days for definite antibiotics; there were no significant differences between durations of antibiotic use in the two groups.

**Table 1:** Comparison of baseline characteristics of patients with ampicillin-susceptible *Enterococcus faecalis/faecium* bacteremia in the glycopeptide and ampicillin-containing treatment groups

Variables	Ampicillin-containing group (%) (*n* = 56)	Glycopeptide group (%) (*n* = 71)	Total (%) (*n* = 127)	*P*-value
Sex, no (%)				0.523
Female	22 (39.3)	24 (33.8)	46 (36.2)	
Age, years	64.3 ± 13.390	64.3 ± 15.617	64.3 ± 14.620	0.690
Body mass index, kg/m^2^	22.5 ± 3.760	23.6 ± 4.157	23.1 ± 4.005	0.084
Comorbidities, no (%)				
Diabetes mellitus	20 (35.7)	21 (29.6)	41 (32.3)	0.463
Congestive heart failure	1 (1.8)	2 (2.8)	3 (2.4)	1.000
Chronic kidney disease	11 (19.6)	3 (4.2)	14 (11.0)	0.006
Liver disease	4 (7.1)	6 (8.5)	10 (7.9)	1.000
Pulmonary disease	1 (1.8)	2 (2.8)	3 (2.4)	1.000
Cerebrovascular accident	3 (5.4)	8 (11.3)	11 (8.7)	0.344
Malignancy	22 (39.3)	22 (31.0)	44 (34.6)	0.329
Immunocompromised status, no (%)				
Solid organ transplantation	3 (5.4)	1 (1.4)	4 (3.1)	0.320
Neutropenia	0 (0)	2 (2.8)	2 (1.6)	0.503
Chemotherapy	5 (8.9)	15 (21.1)	20 (15.7)	0.061
Steroid	1 (1.8)	5 (7)	6 (4.7)	0.227
Immunosuppressant	3 (5.4)	1 (1.4)	4 (3.1)	0.320
Pitt bacteremia score	0.4 (0.8)	1.5 (1.8)	1.0 (1.5)	< 0.001
Hospital-acquired infection, no (%)	28 (50)	69 (97.2)	97 (76.4)	< 0.001
Primary focus of bacteremia, no (%)				< 0.001
Urinary tract infection	21 (37.5)	11 (15.5)	32 (25.2)	
Intraabdominal infection	15 (26.8)	13 (18.3)	28 (22)	
Catheter-related infection	4 (7.1)	19 (26.8)	23 (18.1)	
Primary bloodstream infection	5 (8.9)	14 (19.7)	19 (15.0)	
Skin and soft tissue infection	0 (0)	5 (7.0)	5 (3.9)	
Bone and joint infection	1 (1.8)	3 (4.2)	4 (3.1)	
Others	10 (16.9)	6 (8.4)	16 (12.6)	
Infection management				
Duration of empirical antibiotics, days	2.88 ± 1.349	2.58 ± 1.078	2.71 ± 1.209	0.153
Duration of definitive antibiotics, days	13.39 ± 12.713	10.93 ± 7.849	12.02 ± 10.308	0.913
Source control, no (%)	26 (46.4)	31 (43.7)	57 (44.9)	0.756

Variables are displayed as mean ± standard deviation, unless otherwise specified

Others: infective endocarditis 9 (7.1%), neutropenic fever 1 (0.8%)

### Clinical outcomes in ampicillin-containing and glycopeptide treatment groups

The clinical outcomes are shown in Table 2. The 28-day mortality rate was 19.7% (14/71) in the glycopeptide group and 3.6% (2/56) in the ampicillin-containing group; the difference in the 28-day mortality between the two groups was significant (*p* = 0.006). In addition, the ICU admission rate in the glycopeptide group was 62%, which was higher than that in the ampicillin-containing group (42.9%). There were no significant differences between the two groups in terms of the 30-day recurrence rate, persistent infection rate, adverse drug reaction events, and hospital days after infection.

**Table 2:** Comparison of clinical outcomes in patients with ampicillin-susceptible *Enterococcus faecalis/faecium* bacteremia by treatment group: glycopeptide and ampicillin-containing groups

Outcomes	Glycopeptide group (*N* = 71)	Ampicillin-containing group (*N* = 56)	Total (*N* = 127)	*P*-value
28-day mortality, no. (%)	14 (19.7)	2 (3.6)	16 (12.6)	0.006
Intensive care unit admission, no. (%)	44 (62)	24 (42.9)	68 (53.5)	0.032
30-day recurrence, no. (%)	3 (4.2)	4 (7.1)	7 (5.5)	0.699
Persistent infection, no. (%)^§^	6 (8.5)	5 (8.9)	11 (8.7)	1.000
Drug adverse event, no. (%)	0 (0)	1 (1.8)	1 (0.8)	0.444
Hospital day after infection, days	26 (13, 51.5)	25 (13, 49.5)	26 (13, 50)	0.707

^§^Bacteremia in 7 days

In multivariable logistic regression analysis, after adjusting for confounding variables such as chronic kidney disease, Pitt bacteremia score, hospital-acquired infection, and catheter-related bloodstream infection as the primary focus of bacteremia, glycopeptide treatment in ampicillin-susceptible *E. faecalis/faecium* bacteremia was associated with a 3.7-fold higher odds of 28-day mortality than with treatment with an ampicillin-containing regimen. However, this association was not significant (95% CI, 0.6–23.6; *p* = 0.163) (Table 3).

**Table 3:** Multivariate logistic regression analysis of factors associated with 28-day mortality in patients with ampicillin-susceptible *Enterococcus faecalis/faecium* bacteremia

Variables	Multivariate analysis		
	aOR	95% CI	*P*-value
Glycopeptides use for definitive therapy (vs. ampicillin-containing regimen)	3.7	0.6–23.6	0.163
Chronic kidney disease (vs. none)	0.8	0.1-8	0.860
Pitt bacteremia score ≥ 1 (vs. < 1)	2.4	0.7–7.7	0.156
Hospital-acquired infection (vs. community-acquired infection)	1.5	0.1–18.2	0.728
CRBSI as a primary focus of bacteremia (vs. others)	1.9	0.5–6.7	0.319

aOR, adjusted odds ratio; CI, confidence interval; CRBSI, catheter-related bloodstream infection

## Discussion

In this retrospective cohort study, we investigated the clinical outcomes of ampicillin-containing regimens compared with glycopeptide antibiotics in the treatment of ampicillin-susceptible *E. faecalis/faecium* bacteremia for over a decade. The higher Pitt Bacteremia Score in the glycopeptide group suggests that physicians tended to prescribe glycopeptides for more severe infections. Consequently, mortality and ICU admission rates were higher in the glycopeptide group than in the ampicillin-containing group. However, after adjusting for confounding variables, including the Pitt Bacteremia Score, glycopeptide use was not associated with improved survival in patients with ampicillin-susceptible *E. faecalis/faecium* bacteremia. Our findings are significant because they provide an opportunity to reduce inappropriate glycopeptide use in the treatment of ampicillin-susceptible *E. faecalis/faecium* bacteremia cases.

There is a lack of studies comparing ampicillin-containing regimens with glycopeptides as effective treatments for ampicillin-susceptible *E. faecalis/faecium* bacteremia. Consistent with our findings, some studies have failed to demonstrate a significant association between ampicillin-containing regimens or glycopeptides and clinical outcomes [6–8]. However, despite failing to demonstrate a difference in mortality, Fletcher et al. suggested that definitive vancomycin therapy is associated with poorer long-term outcomes [7]. Other studies have shown higher mortality in the glycopeptide group [9, 14]. The use of glycopeptides in methicillin-susceptible *Staphylococcus aureus* bacteremia has a higher rate of treatment failure or mortality than anti-staphylococcal beta-lactams, owing to their slow bactericidal activity [15–20]. This discrepancy may be attributed to variations in disease severity, primary focus of infection, and exclusion criteria across the enrolled study populations. However, unless the superiority of glycopeptide is evident, glycopeptide therapy as a second-line treatment option is prudent.

Glycopeptides are empirically prescribed for suspected enterococcal bloodstream infections because of their activity against both ampicillin-resistant and ampicillin-susceptible strains. In our study, 54.1% (732/1353) of enterococcal bacteremia cases were caused by *E. faecium*, and only 12.7% were susceptible to ampicillin. Moses et al. reported that 44.7% (17/39) of ICU enterococcal bacteremia cases were ampicillin resistant [21]. Additionally, our study found that the trend towards a higher proportion of immunosuppressed patients, particularly those undergoing chemotherapy, in the glycopeptide group than ampicillin or ampicillin/sulbactam group, although not statistically significant, may reflect clinicians’ preference for broad-spectrum antibiotics in these patients. Previous studies have reported 30-day mortality rates as high as 18.8–35.5% for enterococcal bacteremia [22–26]. Given such high mortality risks, empiric beta-lactam use may not be favored before the final susceptibilities are known. However, many clinicians continue prescribing glycopeptides after susceptibility results confirm ampicillin susceptibility [27, 28]. Our study also demonstrated that over half of clinicians prescribed glycopeptides in patients with ampicillin susceptible enterococcal bacteremia, even though there was no definite evidence of glycopeptide use, penicillin allergy, or other co-infection.

Numerous studies have demonstrated that maintaining glycopeptide use in such cases does not improve survival and may paradoxically increase mortality risk. Prolonged glycopeptide exposure also poses risks of nephrotoxicity and prolonged hospitalization [7, 29–31]. To shift antibiotic prescription habits, large prospective studies using susceptibility data are needed to confirm the benefits of de-escalating to ampicillin. Such evidence of improved outcomes and reduced toxicities with de-escalation could motivate clinicians to optimize treatment for ampicillin-susceptible enterococcal bacteremia. As a result, the importance of expert consultations and interventions in optimizing antibiotic usage will be emphasized.

In our study design, we considered the impact of empirical antibiotic use on patient outcomes. Antibiotic susceptibility results are usually available 48–72 h after blood culture at our institution. However, in some cases, reporting antibiotic susceptibility results is delayed by up to 5 days. Therefore, we excluded cases where empirical antibiotics were used for more than 5 days. Prolonged empirical therapy could significantly influence patient outcomes, potentially confounding our assessment of definite antibiotic efficacy. However, since the empirical antibiotics used for patients in this study were switched to definitive antibiotics within an average of 3 days, it is likely to provide an accurate comparison between the ampicillin-containing group and the glycopeptide group as definitive therapy for ampicillin-susceptible *E. faecalis/faecium* bacteremia.

This study has several strengths, including the exclusion of patients with polymicrobial bacteremia to reduce confounding factors, the evaluation of 28-day mortality outcomes, and the 10-year study duration, which allowed for the examination of temporal epidemiological trends. Multivariate regression analysis adjusted for key confounders, such as severity of illness, infection source, and patient characteristics, is also a strength. Although the study’s subset of patients with ampicillin-susceptible *E. faecium* bacteremia was relatively small, limiting its generalizability to this specific population, this study contributes valuable real-world data on antibiotic selection for the more prevalent cases of ampicillin-susceptible *E. faecalis* bacteremia. However, the retrospective design is inherently susceptible to selection bias, particularly because many patients lacking medical records were excluded, along with unmeasured confounding factors. The modest sample size might have limited the statistical power to detect significant differences. Additionally, granular data on source control measures, such as device or catheter removal, were not captured, which could influence the outcomes, especially given the higher proportion of relatively difficult-to-control intra-abdominal infections in the ampicillin group. Furthermore, our study did not analyze the specific dosing regimens of antibiotics used, which could potentially have affected the interpretation of our treatment outcome comparisons. Despite these limitations, this study provides insights into reducing inappropriate glycopeptide use for treating ampicillin-susceptible enterococcal bacteremia.

## Conclusions

In conclusion, in this study, we demonstrated that the use of glycopeptide antibiotics did not result in superior 28-day mortality outcomes compared with ampicillin-containing regimens for the treatment of ampicillin-susceptible enterococcal bacteremia. Promoting antibiotic stewardship through the judicious selection of narrower agents, such as ampicillin, when susceptible, could reduce unnecessary glycopeptide exposure and preserve this critical broad-spectrum antibiotic. Larger prospective multicenter studies are required to validate the applicability of our results across diverse patient populations and institutional settings.

## Data Availability

All data generated or analyzed during this study are included in this published article and its supplementary information files.

## Abbreviations

ICU: Intensive care units
IRB: Institutional Review Board
MIC: Minimum inhibitory concentrations
VRE: Vancomycin-resistant enterococci
